# Statistics of localized phase slips in tunable width planar point contacts

**DOI:** 10.1038/srep44569

**Published:** 2017-03-16

**Authors:** Xavier D.A. Baumans, Vyacheslav S. Zharinov, Eline Raymenants, Sylvain Blanco Alvarez, Jeroen E. Scheerder, Jérémy Brisbois, Davide Massarotti, Roberta Caruso, Francesco Tafuri, Ewald Janssens, Victor V. Moshchalkov, Joris Van de Vondel, Alejandro V. Silhanek

**Affiliations:** 1 Experimental Physics of Nanostructured Materials, Q-MAT, CESAM, Université de Liège, B-4000 Sart Tilman, Belgium; 2 INPAC-Institute for Nanoscale Physics and Chemistry, Department of Physics and Astronomy, KU Leuven, Celestijnenlaan 200D, B-3001 Leuven, Belgium; 3 Dipartimento di Ingegneria Industriale e dell´ Informazione, Università degli Studi della Campania Luigi Vanvitelli, I-81031, Aversa, Ce, Italy; 4 CNR-SPIN UOS Napoli, Monte S.Angelo-via Cinthia, I-80126, Napoli, Italy; 5 Dipartimento di Fisica “E. Pancini”, Università degli Studi di Napoli ’Federico II’, Monte S.Angelo, I-80126 Napoli, Italy; 6 Laboratory of Solid State Physics and Magnetism, KU Leuven, B-3001, Leuven, Belgium

## Abstract

The main dissipation mechanism in superconducting nanowires arises from phase slips. Thus far, most of the studies focus on long nanowires where coexisting events appear randomly along the nanowire. In the present work we investigate highly confined phase slips at the contact point of two superconducting leads. Profiting from the high current crowding at this spot, we are able to shrink *in-situ* the nanoconstriction. This procedure allows us to investigate, in the very same sample, thermally activated phase slips and the probability density function of the switching current *I*_sw_ needed to trigger an avalanche of events. Furthermore, for an applied current larger than *I*_sw_, we unveil the existence of two distinct thermal regimes. One corresponding to efficient heat removal where the constriction and bath temperatures remain close to each other, and another one in which the constriction temperature can be substantially larger than the bath temperature leading to the formation of a hot spot. Considering that the switching current distribution depends on the exact thermal properties of the sample, the identification of different thermal regimes is of utmost importance for properly interpreting the dissipation mechanisms in narrow point contacts.

Small physical systems behave differently than their bulk counterparts partly due to the increased effect of fluctuations at low dimensions. In the extreme case of one-dimensional structures, long-range order and phase transitions may even be fully suppressed^1^,^2^. Superconducting systems, where the long-range correlation is given by the phase coherence of the entire condensate, are no exception in this respect. Indeed, in a superconducting nanowire with cross section *S* < *ξ*^2^, where *ξ* is the superconducting coherence length, phase fluctuations of multiples of 2*π* prevent the formation of persistent currents and thus menace the overall superconducting state. These phase fluctuations *δφ*, known as phase-slips^3^ (PS), can be triggered either by thermal excitations over^4^,^5^ or by quantum tunnelling through^6^,^7^ an energy barrier Δ*F*. Therefore, according to the Gor’kov-Josephson relation^8^ 2 *eV* = *ħd(δφ*)/*dt*, they give rise to a finite voltage drop *V* even at the lowest temperatures. At zero bias current, a symmetric distribution of 2*nπ* and −2*nπ* events is expected. However, as soon as a finite electric current *I* circulates through the nanowire, one of the two PS events will be favoured due to the decrease of its activation barrier by an amount Φ_0_*I*^9^,^10^. The life cycle of a PS consists of a transformation of all the supercurrent (i.e. charge carriers are Cooper pairs) into normal current (i.e. charge carriers are quasiparticles), thus leading to power dissipation. If heat removal is very efficient or the nanowire’s specific heat is large, the temperature rise at the core of the PS is insignificant and these phase fluctuations can be considered as having a negligible time-correlation, i.e. the development of one event at a time *t* does not favour a second event at the same spot at a later time *t*′ > *t*. For a sufficiently large current, the height of the effective barrier approaches zero, and a series of PS events regularly spaced in time is triggered thus leading to the formation of a persistent voltage step *V* < *R*_*N*_*I*, where *R*_*N*_ is the normal state resistance of the nanowire. In long nanowires, where several PS can coexist, as *I* is further increased, the appearance of a new PS is accompanied by an additional voltage step^11^.

Real systems possess certain thermal inertia implying a finite lifetime *τ* ~ 0.1 ns of the footprints left by a phase slip. This lifetime is linked to the heat diffusion time, which is substantially longer than the order parameter variation and the quasiparticle thermalization time *τ*_*E*_^22^. Giving the fact that depleting the order parameter at an already weakened position is energetically favourable, inefficient heat removal brings about time-correlation between consecutive events. Once the mean delay between consecutive PS events becomes shorter than *τ*, a cumulative heating occurs. As a result, an avalanche of events is triggered leading to a noticeable increase of the local temperature at the constriction, known as hot spot. The most prominent consequence of this effect is a sudden transition to the normal state (*V* = *R*_*N*_*I*) allowing one to define a switching current *I*_sw_ as the current at which the dissipationless state is lost and which is typically determined by a predefined voltage criterion.

Due to the randomness of the activation processes, *I*_sw_ exhibits a statistical distribution with mean value 〈*I*_sw_〉 and standard deviation *σ* depending on *τ*. If heat removal is absent (*τ* → ∞), it may happen that a single event is able to trigger a thermal runaway. In this case the width of the distribution reflects the stochasticity of individual events. In the opposite scenario of very efficient heat removal (*τ* → 0), a detectable voltage jump is only possible after a train of closely packed events. Now, the switching current approaches the critical current of the nanowire (i.e. the depairing current) and the width of the distribution tends to zero. An in-depth theoretical analysis of this phenomenon for the case of long nanowires has been reported in refs 10,12 and for Josephson junctions in ref. 13. Besides the time scales imposed by the rate of PS events Γ, and the heat evacuation time *τ*, there is also an additional time scale imposed by the sweeping rate of applied current. If we were able to ramp up the current with an infinite rate, i.e. much faster than the attempt frequency of single events, we should also attain the critical current of the system^14^. In this limit, of course, the information concerning the activation mechanism is lost. In contrast to that, the statistical distribution of *I*_sw_ at finite sweep rates encodes information about the main physical mechanism triggering the events and whether it is of quantum or thermal origin.

Thus far, most of the investigations have focussed on long nanowires^15^,^16^ where the loci of the events along the nanowire are undetermined, or on short suspended nanowires with poor heat removal conditions^6^,^17^,^18^ where single PS events may have more dramatic consequences. In the present report, we explore the opposite situation of a point contact between two Al superconducting banks forcing the appearance of PS to be highly localized in space. In addition, with the Al film being deposited on a Si/SiO_2_ single crystal wafer, heat removal is rather efficient. The possibility to narrow down the size of the constriction *in*-*situ* through controlled migration of atoms, allows us to switch, on the very same sample, between strong and weak self-heating regimes after the switching process. Furthermore, an analysis of the statistical distribution of *I*_sw_ for different temperatures reveals that the main dissipative mechanism can be ascribed to a train of thermally driven PS events. The physics addressed in the present manuscript might provide a new approach for understanding the statistics of flux avalanche triggering of thermomagnetic origin in thin superconducting films or the escape distribution for magnetization reversal in small particles, although sampling events distribution might be harder in these systems.

## Results and Discussion

### Evidence of weak to strong self-heating transition

**Figure 1:**
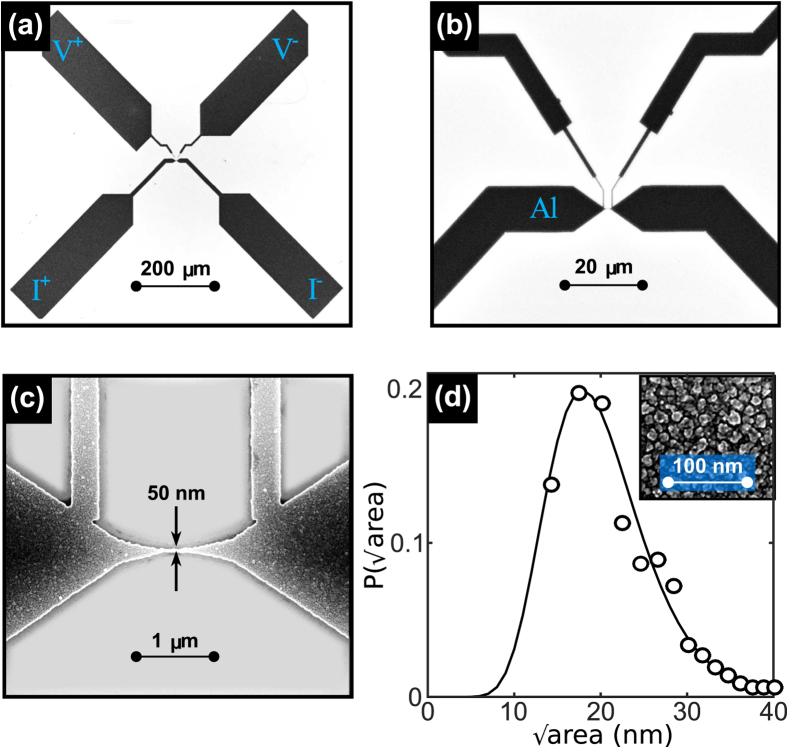
Sample layout. (**a**–**c**) Scanning electron microscopy images of the investigated samples obtained for three different magnifications covering two orders of magnitude on the spatial scale. *V*^+^ and *V*^−^ marks on panel (**a**) denotes the voltage pads with associated polarity, while *I*^+^ and *I*^−^ denotes the pads used to inject current. (**d**) Probability distribution of the grain size displayed with a log-normal fitting. The inset shows a scanning electron microscopy image of the Al surface close to the constriction evidencing the polycrystallinity of the material.

Scanning electron micrographs showing the sample layout are presented in Fig. 1(a–c) for three different magnifications covering two orders of magnitude on the spatial scale. Details of the sample fabrication can be found in Methods. The 25 nm thick Al film shows a clear contrast with respect to the substrate. Current-carrying wires can be readily identified since they are wider than the voltage probes. The granular structure of the Al film is visible in the highest magnification image, Fig. 1(c) and in the inset of Fig. 1(d) evidencing the polycrystallinity of the Al film. The probability distribution of grain size is shown in the main panel of Fig. 1(d) with a log-normal fitting. Note that the starting size of the constriction is larger than the mean grain size. The rather sharp decrease of the nanowire’s width following a nearly hyperbolic shape ensures high probability that PS will be localized in between the voltage probes due to the fact that the number of PS events decreases exponentially as the wire cross section increases.

The samples are highly sensitive to electrostatic discharges and special care has to be taken during bonding, transport, installation and testing (see Methods). Measurements were conducted in two ^3^He cryostats with 300 mK base temperature with proper high-frequency noise filtering, as described in the Methods section. The matching of results from both cryostats is almost perfect. A preliminary investigation of the temperature dependence of the resistance, giving evidence of the transition from thermally driven to quantum excited phase slips when shrinking the constriction below a critical size ~12 nm via electromigration (EM), has been reported in ref. 19. The present work focusses on the analysis of the stochastic nature of phase slip processes and the influence of thermal removal efficiency at the constrictions. We have investigated several samples, with minor adjustments to the exact geometry, and all of them exhibit qualitatively similar results. For the sake of clarity we will report here the results obtained on three of those samples labelled as S1, S2 and S3.

**Figure 2:**
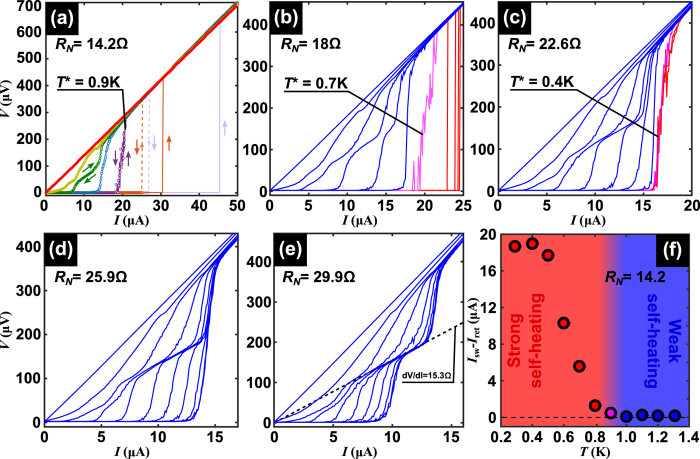
Voltage-current characteristics demonstrating a weak-to-strong self-heating transition. Panels (a–e) show DC current-voltage characteristics for several temperatures ranging from 1.3 K (leftmost curve) to 0.3 K (rightmost curve) with a step of 100 mK for sample S1. For clarity, only curves taken at temperatures of 1.3 K, 1.2 K, 1.1 K, 1 K, 0.9 K, 0.7 K and 0.3 K have been shown in panel (a). Panel (a) corresponds to the virgin sample whereas panels (b) to (e) correspond to the same sample after consecutive reduction of the constriction size via electromigration (as evidenced by the normal state resistance *R*_*N*_). Abrupt jumps of the voltage at low temperatures are indicative of self-heating effects, whereas intermediate voltage plateaus develop in a more isothermal regime (i.e. low self-heating). The transition between both regimes is characterized by a noisy response (magenta line) taking place at a temperature T*. The plateau corresponds to a resistance roughly half of the normal state resistance as shown in panel (e). The *V(I*) curves in the strong self-heating regime are irreversible and become reversible in the weak self-heating regime as shown by the arrows in panel (a). Panel (f) shows the irreversibility parameter *I*_sw_ − *I*_ret_, the difference between respectively the switching and the retrapping current, as a function of temperature, for the virgin sample (*R*_*N*_ = 14.2 Ω).

A typical set of voltage-current (*V* − *I*) characteristics obtained in current driven DC mode for several temperatures and at zero applied magnetic field, are shown in Fig. 2(a). The low temperature response exhibits an abrupt transition from the superconducting state at low bias currents to the normal state at certain switching current *I*_sw_(*T*). At this current, the lifetime of the PS footprints becomes longer than the time elapsed between consecutive PS events and a cumulative heating load triggers the transition to the normal state. In other words, the phase slip is a precursor of a hot spot and therefore, the temperature at the constriction undergoes a strong increase when switching from the non-dissipative to the dissipative branches of the *V(I*) curve. At higher temperatures, the combination of a more efficient heat removal together with a reduced heating power as a consequence of a lower critical current and a higher thermal capacity, allows the phase slips to be present without necessarily transforming themselves into a hot spot. This leads to a resistance plateau above the switching current resembling those well documented in long superconducting whiskers^11^. The fact that the differential resistance of this plateau nearly coincides with half of the normal state resistance, arises from the time averaging of the dissipation during the acceleration/quench cycle of a PS at the constriction, similarly to the process described by the Skocpol-Beasley-Tinkham model^20^. Note that the formation of a PS at the center of the constriction will perturb the condensate on a scale length ~2Λ_*Q*_ much larger than the distance *d* between voltage probes, where Λ_*Q*_ ≈ 5 *μ*m is the charge imbalance relaxation length for Al^21^. Therefore, we can rule out the possibility that the voltage plateau could be ascribed to a normal state switching of a portion (smaller than the distance between voltage contacts) of the constriction.

The transition from weak to strong self-heating after switching is a natural consequence of the steady-state heat balance equation^22^. On the one hand, the heat generation *P*_*in*_ = *ρ(J, T)J*^2^ by the current density *J* traversing a section of material with resistivity *ρ(J, T*), heats up the nanowire. On the other hand, there is a heat removal *P*_*out*_ = *h(T*)(*T* − *T*_*B*_)/*r* towards the cryogenic environment at temperature *T*_*B*_, where *h(T*) is the heat transfer coefficient and *r* = *A*/*P*, being *A* the area and *P* the perimeter of the cross section of the sample^22^. The ratio 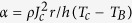, known as the Stekly parameter, accounts for the transition from a weak self-heating regime when 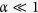 to an important self-heating regime when *α* > 1. In the case where an efficient heat removal is obtained (i.e. high *h* and thus low *α*), the above described plateau in the *V(I*) curves should be also present at lower temperatures.

Possible ways to influence the heat removal could be achieved by changing the substrate material or by immersing the sample in a superfluid ^4^He bath. The former method is unreliable since it implies comparing two different samples, whereas the latter is very limited since only two extreme situations can be contrasted. A more elegant approach for reducing the parameter *α* consists in narrowing down the constriction in a controlled way. By doing so, the critical current decreases and so does the parameter *r* and, as a consequence, a lower power dissipation and a better heat removal are expected. The result of this process, achieved via electromigration (see Methods), is shown in Fig. 2. In the virgin sample (panel (a)), the temperature about which the transition from nearly isothermal to severe self-heating happens is characterized by a noisy response observed at T^*^ = 0.9 K while a clear plateau develops at 1.1 K. As the width of the constriction is progressively reduced (see panels (b)-(e) in Fig. 2), the plateau appears at lower temperatures in agreement with our interpretation of the plateau as a unique signature of an efficient heat removal. It is interesting to point out that the noisy curve results from the fact that the constriction temperature in the steady state is defined by the equation *P*_*in*_(*T*) = *P*_*out*_(*T*) which has at least two *T* values as solution at the transition^22^. As a consequence of this bistability and the switching between these solutions, a noisy response is observed.

Further evidence supporting our interpretation that *V(I*) curves with a single jump to the normal state correspond to strong self-heating effect, whereas curves exhibiting the plateau correspond to a regime where self-heating is unimportant, comes from the irreversible response under reversal of the current sweep^23^. Indeed, in Fig. 2(a) it can be seen that at high bath temperatures, the *V(I*) curves are fully reversible, whereas at low bath temperatures a clear irreversible response develops when sweeping the current up and down. The switching current *I*_sw_ corresponds to the onset of dissipation when increasing current, and the retrapping current *I*_ret_ indicates the transition to the non dissipative regime when decreasing current. Figure 2(f) shows that the irreversibility width *I*_sw_ − *I*_ret_ as a function of temperature starts to grow exactly at the same temperature where the plateau ceases to exist, in agreement with the scenario of a weak to strong self-heating transition. We have also found out that *I*_ret_ is rather independent of temperature, which is consistent with the fact that in the overheated regime, the bath temperature plays a minor role in its determination, as has been already discussed for long nanowires^10^,^24^. We have noticed that different sample batches exhibit the crossover of thermal regimes at different values of the normal resistance of the constriction most likely due to important changes in the heat conductance through the substrate. This fact points out the importance of identifying the crossover temperature T^*^ as that where the plateau in the *V(I*) appears rather than relying on the sample’s normal resistance values.

It is worth mentioning that the here reported transition between thermal regimes of the nanoconstriction is quite similar to the well known thermomagnetic instability observed in superconducting thin films. In the latter, a balance between magnetic and thermal diffusion delimits the transition point^25^.

### Switching current distributions

**Figure 3:**
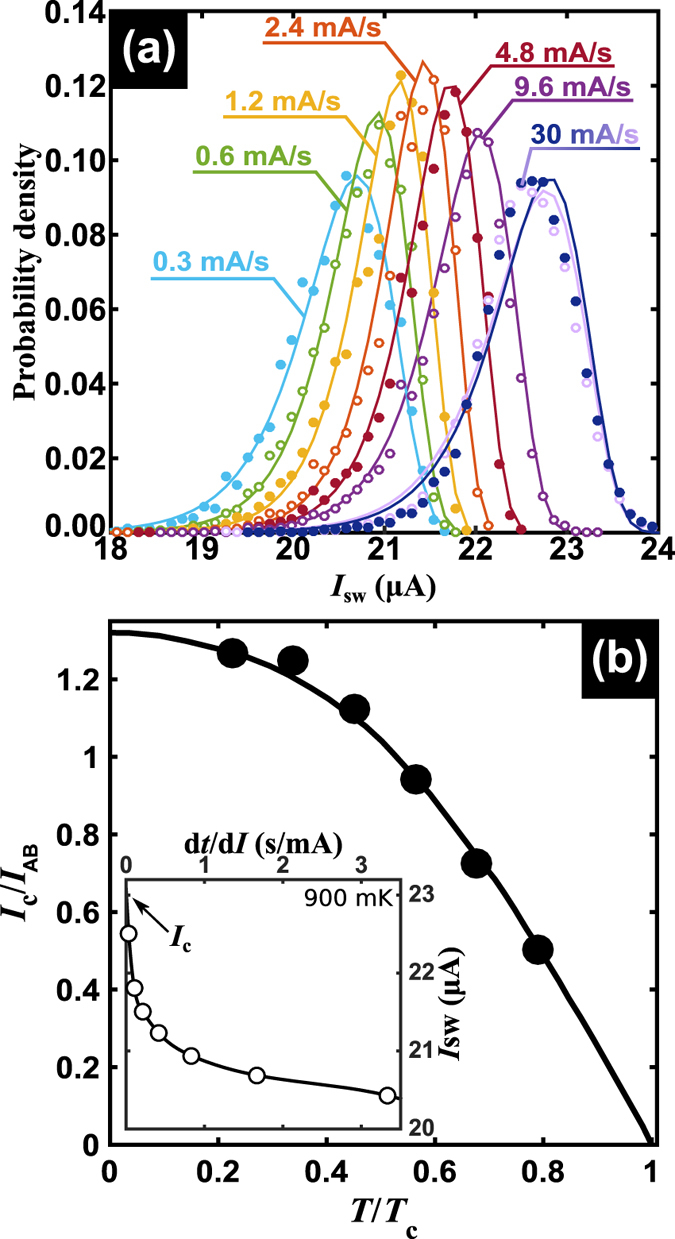
Influence of sweeping rate on the current switch distribution. (**a**) Distributions of switching current for different sweeping rates obtained at *T* = 300 mK for sample S2. Dark blue and light purple distributions (on the extreme right) were taken at the same rate but for different peak amplitude and frequency of sweep. Solid lines are attempts to fit the distributions with an Extreme Value Probability Density (or Gumbel) function. (**b**) Critical current as extrapolated for an infinite rate (*dt*/*dI* = 0), as a function of temperature for sample S3. The continuous solid line corresponds to the fit using the formula of Kulik and Omel’yanchuk, as explained in the text. The normalization factor *I*_AB_ = *π*Δ/2*eR*_N_ = 33 *μ*A corresponds to the Ambegaokar-Baratoff expression of the maximum current for a tunnel junction. An example of extrapolation using splines is shown in the inset for *T* = 900 mK. The size of the experimental point exceeds the error bars as estimated from the difference between a linear and a parabolic extrapolation.

Due to the stochastic nature of the process triggering the switching events, repeating the same experiment under exactly the same external conditions of field and temperature does not necessarily provide the same value of *I*_sw_(*T*). Instead, the obtained *I*_sw_ will be distributed around a mean value 〈*I*_sw_〉(*T*). In order to map the functional form of this statistical distribution, a large number (10^4^) of *V(I*) curves needs to be measured. This can be achieved in a reasonable time when the current sweep rate is high enough. However, as has been pointed out above, the statistical distribution depends on the chosen current sweep rate^14^. This is illustrated in Fig. 3(a) where several distributions obtained for different current ramps have been obtained on the same (non electromigrated) sample at 300 mK. The continuous curves shown in Fig. 3(a) correspond to fits using the Gumbel function as described in Methods. The switching current was determined using a criterion of 33% of the voltage jump. Since the number of experiments is constant, so is the area under each distribution curve. As explained in ref. 14 the vertical axis on Fig. 3(a) corresponds to the probability density of switching *P(I*_sw_) multiplied by the bin size Δ*I* = 100 nA.

In the present case a triangular shape sweeping function has been used with a constant maximum current of 30 *μ*A. It can seen in Fig. 3(a) that increasing the rate leads to an increase of 〈*I*_sw_〉, as explained before^14^. However, unlike in the uncorrelated PS model where a continuous narrowing down of the distribution is expected^14^, we now observe that the standard deviation of the distribution follows a non-monotonic trend. A possible explanation for the widening of the distribution when increasing the rate is the fact that as 〈*I*_sw_〉 increases, heat dissipation also increases and so does the imprint left by PS events. Using the reasoning of Shah *et al*.^12^, the smaller the number of phase slips in the sequence inducing the superconducting-to-resistive thermal runaway, the larger the stochasticity in the switching process and, hence, the wider the distribution of switching currents. In other words, as the sweeping rate increases, shorter train of PS events are needed leading to wider distributions. This trend, i.e. an increasing width of the distribution and increase of 〈*I*_sw_〉 as current ramp increases, has been reproduced by Monte-Carlo simulations including self-heating effects (see Methods).

It has been theoretically shown^14^,^26^ that the distribution of switching currents depends on the frequency *f* and amplitude *A* through the sweep rate *fA*. To illustrate this fact, the rightmost curve of Fig. 3(a) shows data corresponding to different values of maximum current (30 and 60 *μ*A) and period (500 and 250 Hz) while keeping the same rate of 30 mA/s.

When the sweeping rate is increased to infinity, the critical (depairing) current of the nanoconstriction *I*_c_ should be reached. Although an infinite sweeping rate can not be achieved experimentally, it is possible to estimate this value by extrapolating the data corresponding to finite sweeping rate as shown in the inset of Fig. 3(b) for *T* = 900 mK. The main panel of Fig. 3(b) shows *I*_c_(*T*) determined by repeating the same extrapolation procedure for several temperatures. Note that the particular geometry of the used nanoconstriction could be regarded as a short nanowire (also known as S-c-S junction) or rather as a diffusive S-N-S junction^27^. For the former, Kulik and Omel’yanchuk^28^,^29^ have provided a solution from the microscopic theory for the critical current for superconductor-constriction-superconductor junctions at arbitrary temperatures. This prediction is compared to the extrapolated points in Fig. 3(b). The normalization factor *I*_AB_ = *π*Δ/2*eR*_N_ = 33 *μ*A corresponds to the Ambegaokar-Baratoff expression of the maximum current for a tunnel junction. As we suggested above, our samples could also be regarded as diffusive S-N-S microbridges. A detailed analysis of these systems can be found in ref. 27 and the references therein. These authors show that for values of the ratio *ε*_c_/Δ ≫ 1 (short junctions limit), where *ε*_c_ = *hD*/2*πL*^2^ is the Thouless energy scale, and Δ is the superconducting energy gap, the Kulik-Omel’yanchuk formula is recovered. This limit is satisfied in our samples for which we find *ε*_*c*_/Δ ~ 23.

**Figure 4:**
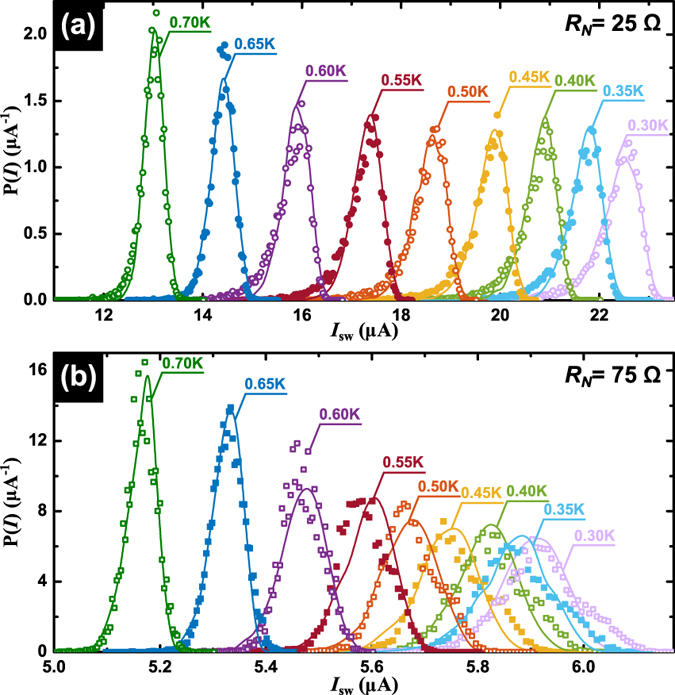
Switching current distributions. Temperature scan of switching current distributions obtained at a rate of 1.2 mA/s, (**a**) before and (**b**) after electromigration for sample S2. At each temperature, ten thousands *V(I*) and the corresponding values of *I*_sw_ are recorded. Solid lines are attempts to fit the distributions with Monte-Carlo simulations including the effects of self-heating by multiple PS.

As pointed out before, the limiting case of infinite sweeping rate pushes *I*_sw_ towards *I*_c_ and therefore conceals the physics of phase slip events. This has been addressed in ref. 17 where the temperature dependence of *I*_sw_ in Mo_3_Ge nanowires obtained at finite sweeping rates exhibits a change of the functional dependence which was attributed to the transition from thermally activated phase slips to the macroscopic quantum tunneling of the superconducting phase. Motivated by this approach, we have investigated the switching current distributions at a finite rate of 1.2 mA/s. Figure 4(a) summarizes the obtained switching current distributions for the virgin sample at several bath temperatures, whereas panel (b) of this figure shows the distributions for the same sample after narrowing down the constriction in such a way that resistance increased by a factor of three. Assuming that the electromigration takes place in a single spot at the narrowest point of the isthmus^30^, this change of resistance roughly corresponds to a reduction of the constriction width down to ~17 nm. This width falls above the critical value of ~12 nm where a crossover towards QPS driven dissipation has been reported for similar samples^19^ suggesting that in the present samples, TAPS should be the dominant mechanism. As recently reported in ref. 19, further electromigrating the sample allows one to access the QPS regime where a critical current becomes negligible, therefore preventing the study of the switching statistics.

The continuous lines in Fig. 4 are Monte Carlo fittings to the data taking into account self-heating effects produced by thermally excited PS, following the procedure described by Massarotti *et al*.^13^ and detailed in Methods. These fittings reproduce reasonably well the dependency of the experimental data for the virgin sample, whereas a somewhat less goodness of fit is observed for the electromigrated sample. A careful look to the low temperature distribution in the narrower sample reveals the existence of a second satellite hump next to the main peak which we attribute to the existence of a second competing nucleation point of phase slips events. Indeed, it has been shown that not only the current crowding effects produced by the reduced cross section of the wire lead to electromigrated voids, but that grain boundaries may also serve as nucleation points of this process^31^,^32^. As a consequence, it is likely that the electromigration does not occur exactly at the narrowest part of the bridge, thus giving rise to two weak points with different critical currents^19^. In view of the fact that two contiguous nucleation points will inevitably and mutually infuence each other, the total probability density function cannot just be considered as the product of two individual and independent processes, a problem that has not been addressed so far.

**Figure 5:**
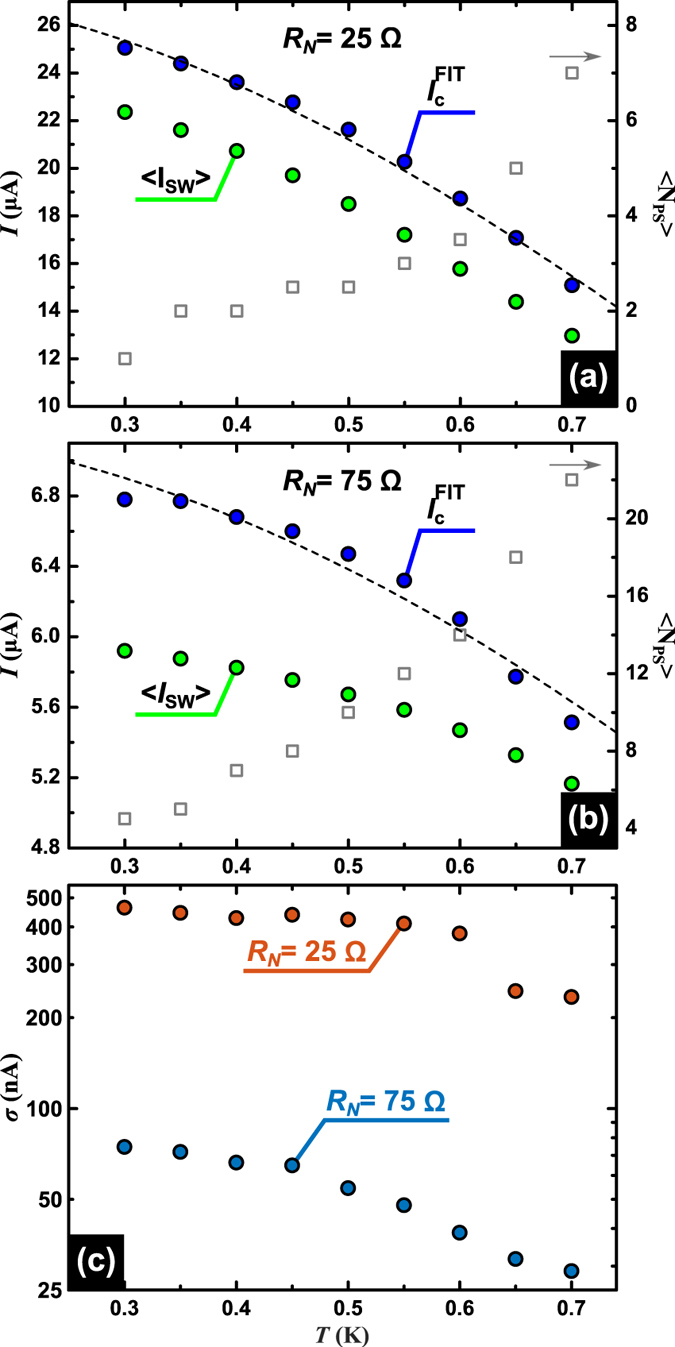
Temperature dependence of the mean switching current and its standard deviation. Green and blue circular symbols are respectively experimental data and values used in the fits presented in Fig. 4 before (**a**) and after (**b**) electromigration. Light grey squared symbols are the mean number of PS involved in the switching. Black dotted lines correspond to Bardeen’s expression for critical current. Panel (c) shows a monotonous decrease of the standard deviation of the switching current distribution.

A direct determination of the mean switching current 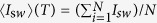 and the standard deviation 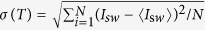 can be obtained from the experimental data shown in Fig. 4. The results are presented in Fig. 5. Panel (a) shows the mean switching current 〈*I*_sw_〉 for the virgin sample, along with the critical current 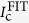 obtained from the fitting and Bardeen’s expression^33^ *I*_c_(*T*) = *I*_c0_(1 − (*T*/*T*_c_)^2^)^3/2^ (dashed line). The modelling considers a bias current *I* ramping from zero to 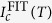. As soon as the temperature of the system reaches a threshold value *T*_th_ the system is defined to be in the resistive state and the relative value of the current is recorded as switching current *I*_sw_. The 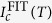 estimated from the simulations and shown in Fig. 5 should be considered as the critical current (at the corresponding bath temperature) in absence of thermal fluctuations. In this sense it is expected and indeed observed in Fig. 5(a,b), that its value becomes larger than the experimentally determined 〈*I*_sw_〉(*T*). Panel (b) shows the same parameters for the sample after electromigration. In both panels, it is also included in the right vertical axis (grey square points) the number *N*_PS_ of PS events needed to trigger the switching process. Notice that for the virgin sample at the lowest measured temperature *T* = 0.3 K, *N*_PS_ = 1 and therefore a single phase slip is sufficient to increase the wire temperature above its critical temperature and trigger the switching process. After narrowing down the constriction (Fig. 5(b)) the switching process takes place at lower currents, as expected. The fact that heat removal has been improved by reducing the constriction also leads to a higher number of PS needed to trigger the switching, consistent with the scenario described above. Furthermore, for both cases (virgin and electromigrated sample), *N*_PS_ monotonously increases with temperature, implying a better heat removal, a weaker heating power and a lower level of stochasticity as *T* increases. This is also in agreement with the monotonous decrease of the standard deviations *σ* of the distributions shown in Fig. 5(c) and the fact that the electromigrated sample exhibits a smaller *σ*.

The reduction of *σ* when decreasing the effective width of the constriction is consistent with previous reports on long nanowires^34^. A decreasing *σ* with increasing *T* at high temperatures has been associated to the need of a train of phase slip events before switching to the normal state, whereas the observed flatting down of *σ* at low temperature has been interpreted as a signature of macroscopic quantum tunneling^34^. In order to understand the change of *σ* with temperature for the particular case of a nanoconstriction, it is necessary to take into account the interplay of several competing effects. On the one hand, as *T* diminishes, the rate of thermally activated phase slips decreases, fluctuations weaken and consequently *σ* should also decrease. On the other hand, lowering *T* leads also to an increase of the mean value of switching current (〈*I*_sw_Δ〉) and a decrease of thermal conductivity (*κ*) and heat capacity (*c*) of the superconductor, all three factors combining to make the phase slips more effective at heating the wire which would broaden the distribution. Our experimental data confirms that the influence of the last three factors overruns the influence of the decrease of thermally activated phase slips when temperature is lowered, hence the broadening observed for the distribution^10^,^18^.

**Figure 6:**
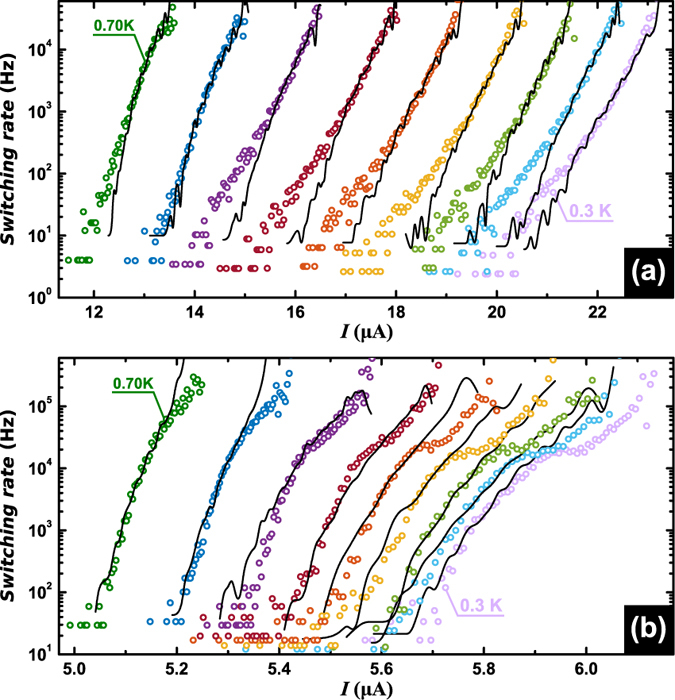
Switching rates from the non-dissipative to the dissipative states. Before (**a**) and after (**b**) narrowing down the constriction via electromigration. Experimental data corresponds to bath temperatures between 0.7 K (left most) and 0.3 K (right most). The black lines correspond to Monte-Carlo simulations for multiple Phase Slip events, including self-heating effects.

Let us now determine experimentally the switching rate Γ(*I, T*). Since *dI*/*dt* is constant and *P(I*) is obtained experimentally, Γ(*I, T*) can be deduced by discretization (see Methods), from the experimental data shown in Fig. 4. If we denote *K* = 1 the channel corresponding to the highest value of *I*_sw_ in the distribution, then Γ(*K*) can be computed according to the formula^35^:


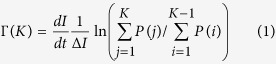


where Δ*I* is the chosen binning size. The results of this analysis have been summarized in Fig. 6 where the experimental data is indicated with symbols and continuous lines correspond to the theoretical fittings obtained from the Monte Carlo simulations including self-heating effects. The noisiness of the theoretical curves arises from the finite number of runs (4000) used to determine the distribution. At this point, we should stress that macroscopic quantum tunnelling does not need to be invoked to explain the observed effect at low temperatures in this particular sample. As recently reported in ref. 16, it is possible to access the QPS regime by further electromigrating the sample. In this case, however, the critical current approaches zero, therefore making difficult to find a suitable range of samples’ widths where it is possible to evidence the QPS influence on the switching statistics.

## Conclusions

We have identified the presence of two distinct thermal regimes in planar superconducting nanoconstrictions. On the one hand, a weak self-heating regime where the heat removal is rather efficient and the superconducting constriction switches to the normal state in two steps when increasing current. On the other hand, a strong self-heating regime where the transition to the normal state corresponds to a single abrupt jump. In the former regime, a phase slip can exist as an oscillating order parameter with periodic phase jumps, whereas in the latter a hot spot develops. We have demonstrated the thermal origin of this transition by shifting it when reducing the size of the constriction. Irrespective of the thermal regime developed at currents larger than the switching current, the distribution of switching currents can be well accounted within a purely thermally activated phase slips scenario. In addition, this work points out to the importance of extending the current theoretical model to include mutual interaction of phase slip events occurring at short distances.

Although this work provides the first step towards an in-depth understanding of the effects of thermal fluctuations, further studies at lower temperatures are needed to clarify the role of quantum phase slips. The possibility to tune in a controlled way the thermal properties of the nanobridge and the fundamental energy scale, by reducing the critical current of the nanobridge through successive electromigration processes, will be the key knob to definitively address the transition to the quantum regime.

## Methods

### Sample fabrication

The Al nanowires were defined by Electron Beam Lithography (EBL) on Si/SiO_2_ substrate (750 ± 50 *μ*m Si, 300 ± 25 nm SiO_2_) covered by a resist mask (double layered, PMMA 950 K, 3.5% solid content in ethyl lactate solvent + PMMA/MA 33%, 4% solid content in methoxy propanol solvent, 150 nm + 200 nm), using nanofabrication system from Raith GmbH. The Si wafer had crystal orientation (100). Subsequently, an Al thin film (~25 nm) was deposited using Molecular Beam Epitaxy (MBE) with deposition rate of 1 Å s^−1^ and pressure in the chamber under 10^−8^ mbar. The deposition was followed by a lift-off process.

### Sample granularity

The Al grain size distribution is represented in Fig. 1(d), where the probability density is plotted as a function of 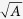, the characteristic grain radius. This distribution was obtained by using the particle analysis tool provided by the ImageJ software, after filtering and binarization of the original image. A threshold was set at 200 nm^2^ on the minimum value of the grain area, in order to avoid artefacts coming from noise in the image. The grains were counted in 50 nm^2^ wide classes according to their area and the distribution was normalized by the total number of grains detected. The line in Fig. 1(d) shows the fitting with a log-normal distribution and gives a mean characteristic grain radius of 18 nm.

### Sample installation and handling

Aluminum transport nanobridges used in this work are very sensitive to electrical discharges. For this reason, wirebonding and installation were performed using a special platform allowing us to short-cut the four pads of the aimed sample and connect them to the reference of the wirebonder. In addition to that, once installed in the cryostat, we used a make-before-break mechanism that allowed to disconnect the four pads of the samples from the ground, then from each other and finally connect them to the instruments for transport measurements. When the measurements are finished, the same procedure is used in reverse order to finally have the four pads of the sample short-cut and connected to the same reference (typically the ground).

### Cryostats and filtering

Measurements were performed in two very similar ^3^He/^4^He Oxford cryostats but with different filtering systems. The first one was equipped of *π*-filters with cut-off frequency of 1 MHz at room temperature while the second one had electromagnetic interference filter at room temperature, RC filters with a cut-off frequency of 1.6 MHz anchored at 1.5 K and a combination of copper powder and twisted pair filters thermally anchored at the mixing chamber of the dilution refrigerator. This latter system is described in ref. 36.

### Switching current distributions

The switching current distributions were taken using a digital phosphor oscilloscope (TDS5032B from Tektronix). A triangular wave signal was applied with desired amplitude and frequency using a function generator (K6221 from Keithley). The response of the sample was acquired via a low noise pre-amplifier (SR560 from Stanford Research Systems) giving an amplified signal to the oscilloscope. Triggering was ensured by a trigger link between function generator and oscilloscope. A home-made software was then in charge of acquiring the data from the oscilloscope: a voltage criterion was set for the switching, then time position of the switching event was located and transformed into the corresponding value of current via the knowledge of the applied wave. Ten thousands values for the switching current were registered for each temperature using this procedure.

### Analytical and numerical fittings

The probability distribution of individual PS events when the current is swept with a rate *dI*/*dt* is given by^35^,^37^,^38^,


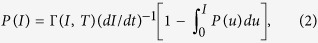


where Γ(*I, T*) is the rate of events. This equation is a particular case of a Volterra equation of the second kind^39^ with general form of the solutions that can be derived analytically^40^:


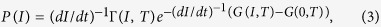


where *G(u, T*) = ∫Γ(*u, T)du*. For a vast diversity of functional forms of Γ(*I, T*) (see refs 10,17,38), Eq. (3) can be approximated by the Gumbel distribution^17^,^34^:


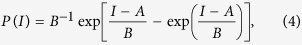


which has a mean 〈*I*_sw_〉 = *A* − *γB*, where *γ* ≈ 0.577 is the Euler-Mascheroni constant, and a standard deviation 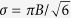. Indeed, linearizing 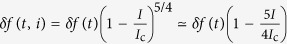, the probability of switching is given by:


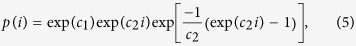


where 
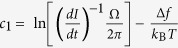
 and 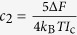. The goodness of fit using this expression is comparable to the one obtained using the Gumbel distribution.

The degree of asymmetry (skewness) and tailness (kurtosis) of the Gumbel distribution are given by the third and fourth moments of the function, respectively, and acquire constant (universal^26^) values. Strictly speaking, Eq. 2 is valid if the temperature *T* is well defined, i.e. if self-heating effects play no role. Since in our case the system is not in equilibrium with the cryogenic environment, it is necessary to take into account the heating driving process as described in refs 10,13. In the proposed model, a sample of length *L* in contact with a thermal bath at the temperature *T*_*b*_ is considered. A PS event induces heating in a segment 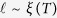. The equation for heat diffusion after the occurrence of a PS is:


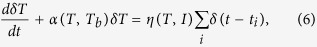


where *δT* = *T* − *T*_*b*_ is the local increase of temperature and *α(T, T*_*b*_)^−1^ is the relaxation time scale for the temperature *T* to return to its equilibrium value *T*_*b*_ after a sudden temperature rise *η* due to the PS at time *t*_*i*_, has occurred. The distributions are obtained by running the simulation with 4000 determinations of the switching current. The numerical fitting procedure is obtained by adjusting two parameters: the critical current 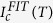 and the threshold temperature *T*_*th*_, i.e. the temperature above which we declare that the system has switched to a finite voltage state, which implies how many heating events are needed to induce the switching. We have found that for all fittings 0.11 K < *T*_*th*_ − *T* < 0.17 K. For the simulations, we have used the following parameters *T*_*c*_ = 1.3 K, diffusion constant *D* = 49 × 10^14^ nm^2^ s^−1^, density of states at the Fermi level *ρ(ε*_*F*_) = 34.4 ev^−1^ nm^−3^. The length of the nanowire was 1500 nm, the length of its central part (along which phase slips may occur) was 400 nm and its resistance in the normal state was 25 Ω. The superconducting coherence length at zero temperature was taken to be equal to 125 nm and the value of the superconducting gap to 2 × 10^−4^ eV.

### Electromigration

**Figure 7:**
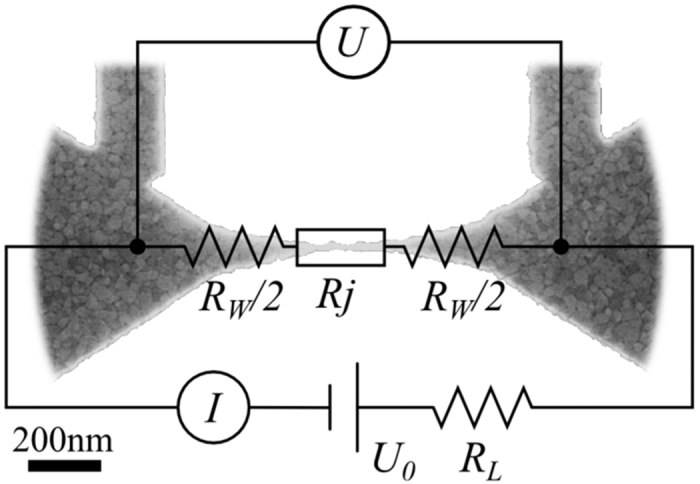
A generalized scheme of the EM setup. Here *U*_0_ is the bias applied to the whole system, *I* is the current in the system, *R*_*L*_ is the leads resistance, *U* is the bias measured on the region where EM is occurring, *R*_*j*_ is the junction resistance and *R*_*W*_ is the line resistance (wires resistance) between the voltage probes besides the junction resistance. Background: SEM image of a sample junction prepared for EM.

EM was conducted by applying a software controlled voltage to the sample. Typical values of current needed to electromigrate the depicted Al nanoconstrictions are on the order of a few mA. As shown in Fig. 7 the EM setup is based on a four point probe measurement scheme. This allows us to directly measure the conductivity of a junction area by using a current source and a voltmeter. In these experiments the control feedback algorithm decides to increase or decrease the applied bias trying to keep the variation of conductance d*G*/d*t* at the value set by an operator. This process continues until the desired resistance of the constriction is reached. A similar method, consisting of high bias voltage pulses, has been used by Aref *et al*. in ref. 41 in order to tune the switching current of a superconducting MoGe nanowire.

## Additional Information

**How to cite this article:** Baumans, X. D.A. *et al*. Statistics of localized phase slips in tunable width planar point contacts. *Sci. Rep.* **7**, 44569; doi: 10.1038/srep44569 (2017).

**Publisher's note:** Springer Nature remains neutral with regard to jurisdictional claims in published maps and institutional affiliations.
